# Endothelial Activation: The Ang/Tie Axis in Sepsis

**DOI:** 10.3389/fimmu.2018.00838

**Published:** 2018-04-24

**Authors:** Aleksandra Leligdowicz, Melissa Richard-Greenblatt, Julie Wright, Valerie M. Crowley, Kevin C. Kain

**Affiliations:** Sandra Rotman Centre for Global Health, University Health Network and University of Toronto, Toronto, ON, Canada

**Keywords:** endothelial dysfunction, Tie2 receptor, angiopoietins, sepsis, malaria, critical care

## Abstract

Sepsis, a dysregulated host response to infection that causes life-threatening organ dysfunction, is a highly heterogeneous syndrome with no specific treatment. Although sepsis can be caused by a wide variety of pathogenic organisms, endothelial dysfunction leading to vascular leak is a common mechanism of injury that contributes to the morbidity and mortality associated with the syndrome. Perturbations to the angiopoietin (Ang)/Tie2 axis cause endothelial cell activation and contribute to the pathogenesis of sepsis. In this review, we summarize how the Ang/Tie2 pathway is implicated in sepsis and describe its prognostic as well as therapeutic utility in life-threatening infections.

## Introduction

Sepsis is a state of life-threatening organ dysfunction caused by a dysregulated host response to infection (1). Despite being a leading cause of global morbidity and mortality, sepsis has no known specific therapies (2). The current critical illness classification defines organ dysfunction by an increase in the Sequential [sepsis-related] Organ Failure Assessment (SOFA) score (3). However, sepsis is a heterogeneous syndrome that is not completely characterized using non-specific clinical variables. The use of generic classification models for complex, critically ill patients may impede appropriate triage and management and limit the application of personalized treatment strategies (4). A more comprehensive characterization of sepsis pathophysiology may reveal new opportunities for precision medicine-based therapies with either novel or repurposed agents that target specific pathways contributing to disease (4, 5).

Microvascular dysfunction is the endpoint of many life-threatening infections (6), and a well-established relationship exists between endothelial injury and sepsis (7–9). Determining the severity of microbial infections is challenging early in the course of disease when clinical scoring systems have limited prognostic utility. Many classic markers of end-organ compromise (such as serum lactate, bilirubin, and creatinine clearance) are not informative until significant clinical deterioration has occurred. However, the timely recognition of sepsis is critical as early aggressive management can considerably reduce morbidity and mortality (10). Early prognostic indicators of critical illness severity are needed to improve early recognition, appropriate triage, management, and outcomes, as well as to enable rational health resource allocation. This review examines the role of the angiopoietin (Ang)/Tie2 axis in sepsis and summarizes its potential applications in the early recognition of sepsis and as a therapeutic target to improve clinical outcomes.

## The Ang/Tie Axis in Sepsis

Vascular function and permeability are regulated by endothelial-specific receptor tyrosine kinases and their ligands, including the vascular endothelial growth factor (VEGF)-VEGF-receptors (VEGFRs), and the Ang-Tie receptors. The Tie1 and Tie2 receptors constitute the Tie receptor family and are almost exclusively expressed in the endothelium (11, 12). Tie2 functions as a receptor for the Ang family of proteins (Ang1, Ang2, and Ang4), while Tie1 is an orphan receptor that can be activated by Angs *via* its interaction with Tie2 (13). Binding of Angs to Tie2 in the stable vasculature promotes the formation of a Tie1/Tie2 heterodimer in a β_1_ integrin-dependent manner, resulting in Tie2 trafficking to cell–cell junctions (14, 15).

**Figure 1:**
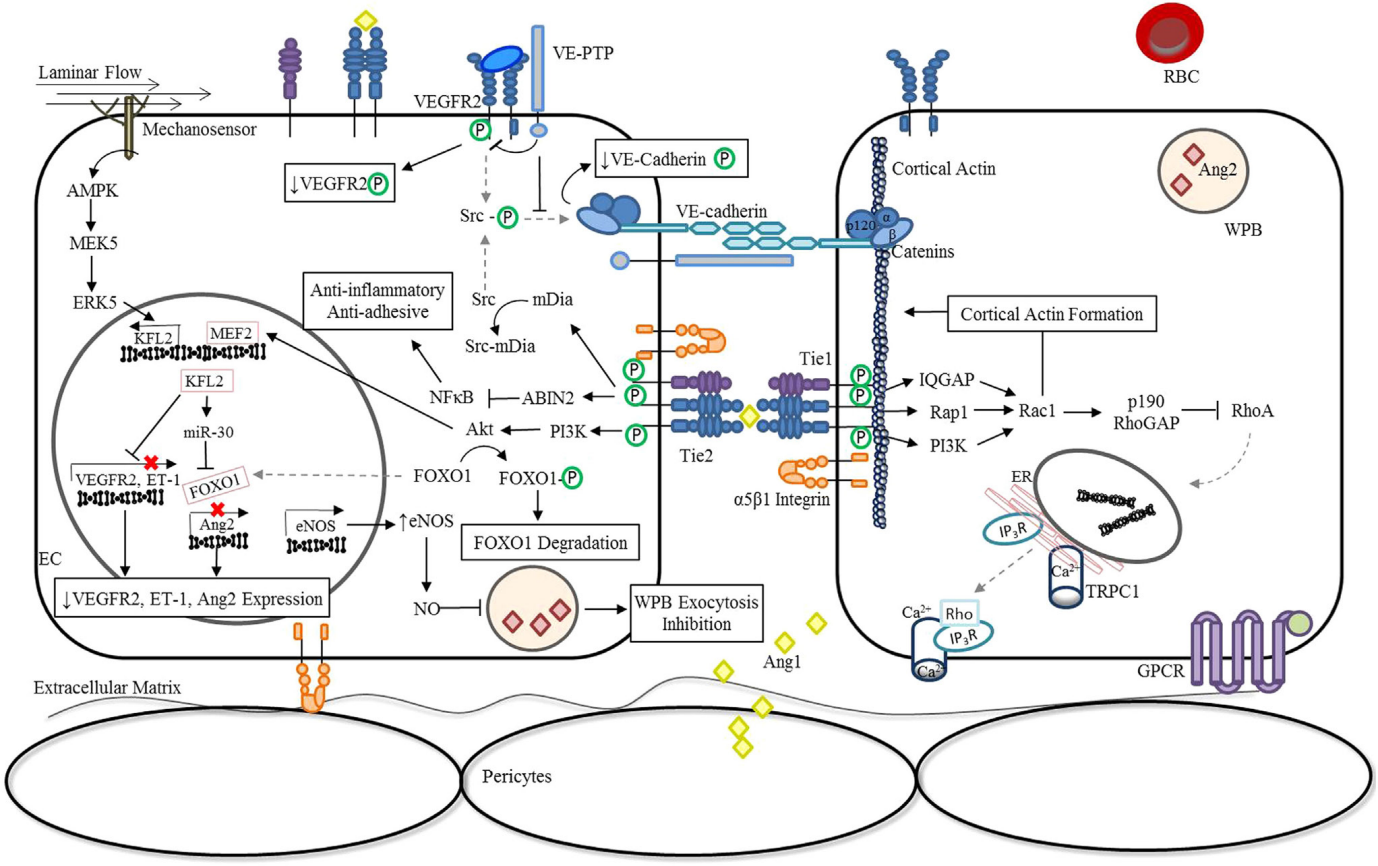
Ang/Tie pathway regulation of vascular stability. In the absence of inflammation or infection (stable vasculature), Ang1 is secreted by mesenchymal cells (22, 23) and low levels of autocrine Ang2 are constitutively secreted (24). Binding of Angs to Tie2 promotes the interaction of Tie1 and Tie2 in a β_1_ integrin-dependent manner and regulates Ang-induced Tie2 trafficking to cell–cell junctions (14, 15). Under these conditions, endothelial Tie1 enhances the activity of Ang1 and is essential for Ang2 agonistic activity (14, 15). Oligomerized Ang1 also induces the translocation of Tie2 to cell–cell contacts and induces an Ang1-bridged Tie2 *trans*-association (17). The resulting phosphorylation of Tie2 leads to the activation a number of downstream signaling pathways that are involved in vessel stability and endothelial barrier function. Activation of the downstream serine kinase, AKT, leads to the phosphorylation and nuclear exclusion of FOXO1, and repression of its target genes which include Ang2 (18–20). Ang1 inhibition of NF-κB reporter gene activity *via* activation of ABIN2 dampens the expression of adhesion molecules and pro-inflammatory cytokines (25, 26), preventing further activation of the endothelium through localized inflammatory mediators. In parallel, Ang/Tie2 signaling stimulates the transcriptional activity of MEF2 through the PI3K/AKT pathway to induce the expression of a second transcription factor KFL2 to ultimately counteract VEGF-mediated vascular permeability (↑eNOS expression; ↓VEGFR2 and ET-1 expression) (27). The increase in NO generated by eNOS combined with the negative regulation of Ang2 expression during quiescence significantly reduces luminal concentrations of Ang2 (28). In addition, KFL2 induces miR-30 expression, further blocking the transcription of Ang2 (29). The phosphorylation of Src, which generally culminates in the phosphodependent internalization of VE-cadherin, is also inhibited by Ang1/Tie2. Signaling through Ang1 leads to the activation of mDia, resulting in the sequestration of Src, and prevention of subsequent phosphorylation by VEGFR2 (30). At cell–cell junctions, Ang1/Tie2 also blocks VEGF signaling by promoting the interaction of VEGFR2 with VE-PTP (31). Lastly, activation of Tie2 can lead to the activation of the GTPase, Rac1, *via* IQGAP (32), Rap1 (33), or PI3K/Akt (34)-dependent pathways to stabilize the cortical actin cytoskeleton and maintain adherens and tight junctions between cells (35). In the presence of LPS, activation of the RhoA-specific GTPase activating protein, p190RhoGAP, by Rac1, is essential for shifting the balance away from RhoA rearrangement of the actin cytoskeleton and preventing vascular permeability (32, 35). Abbreviations: ABIN2, A20-binding inhibitor of nuclear factor-κB-2; Ang1, angiopoietin-1; Ang2, angiopoietin-2; EC, endothelial cell; eNOS, endothelial nitric oxide synthase; ER, endoplasmic reticulum; ET-1, endothelin-1; FOXO1, forkhead box protein O1; GPCR, G-protein-coupled receptor; IP_3_R, inositol triphosphate receptor; IQGAP, IQ motif containing GTPase activating protein; KLF2, Krüppel-like factor-2; LPS, lipopolysaccharide; mDia, mammalian diaphanous; MEF2, myocyte enhancer factor-2; miR-30, microRNA-30-5p; NF-κB, nuclear factor-κB; NO, nitric oxide; PI3K, phosphoinositide triphosphate kinase; Rac1, RAS-related C3 botulinum toxin substrate 1; Rap1, Ras-related protein 1; RBC, red blood cell; RhoA, Ras homolog gene family, member A; Src, proto-oncogene tyrosine-protein kinase; TRPC1, transient receptor potential channel-1; VE-cadherin, vascular endothelial-cadherin; VEGF, vascular endothelial growth factor; VEGFR2, vascular endothelial growth factor receptor 2; VE-PTP, vascular endothelial protein tyrosine phosphatase; WPB, Weibel-Palade Body; P, phosphorylation.

During vascular quiescence, mesenchymal cells secrete Ang1, a strong Tie2 agonist, to support endothelial survival and vascular stability (16). Under these conditions, oligomerized Ang1 promotes the *trans*-association of Tie2 at cell–cell contacts and can also anchor Tie2 to the extracellular matrix (ECM) through binding fibronectin, collagen, and vitronectin with high affinity (17). In addition to forming adhesive structures between cell–cell and cell–substratum contacts, Tie2 activation by Ang1 induces a number of downstream signaling cascades as shown in Figure 1. Notably, the serine kinase, Akt, is activated and results in the phosphorylation of the Forkhead box protein O1 (FOXO1) transcription factor, leading to the nuclear exclusion of FOXO1 and decreased expression of its target genes (18–20). The inhibition of *Foxo1* transcriptional activity in endothelial cells (ECs) induces expression of genes involved in vessel stability and the repression of genes involved in vascular destabilization, including Ang2. Consequently, during quiescence, Ang2 is constitutively expressed at low levels and co-localizes with von Willebrand factor (vWF) within the Weibel Palade bodies (WPBs) of ECs (21).

Upon stimulation of ECs by inflammatory cytokines or VEGF, Ang2 expression and secretion from WPB are increased, creating an autocrine regulatory mechanism of Tie2 signaling (36, 37). However, in contrast to Ang1, the action of Ang2 on Tie2 signaling has an additional level of complexity that is dependent on the microenvironment of ECs (38–41). While the Tie1/Tie2 heterodimeric complex enables both Ang1 and Ang2 to function as Tie2 agonists (14, 15), in the presence of an infection or inflammation ECs shed the Tie1 ectodomain, and Ang2 binding results in Tie2 antagonism (14). Similarly, Tie1 shedding decreases Ang1 agonistic activity (reduced Tie2 phosphorylation), demonstrating that Tie1 is required for the full activation of Tie2 (14, 15). Taken together, infection increases Ang2 expression and its release from WPBs, tipping the luminal Ang balance in favor of Ang2. Consequently, the increase in Ang2/Tie2 binding, particularly under conditions of enhanced Tie1 shedding, blocks Tie2 activation and contributes to the destabilization of the endothelium.

**Figure 2:**
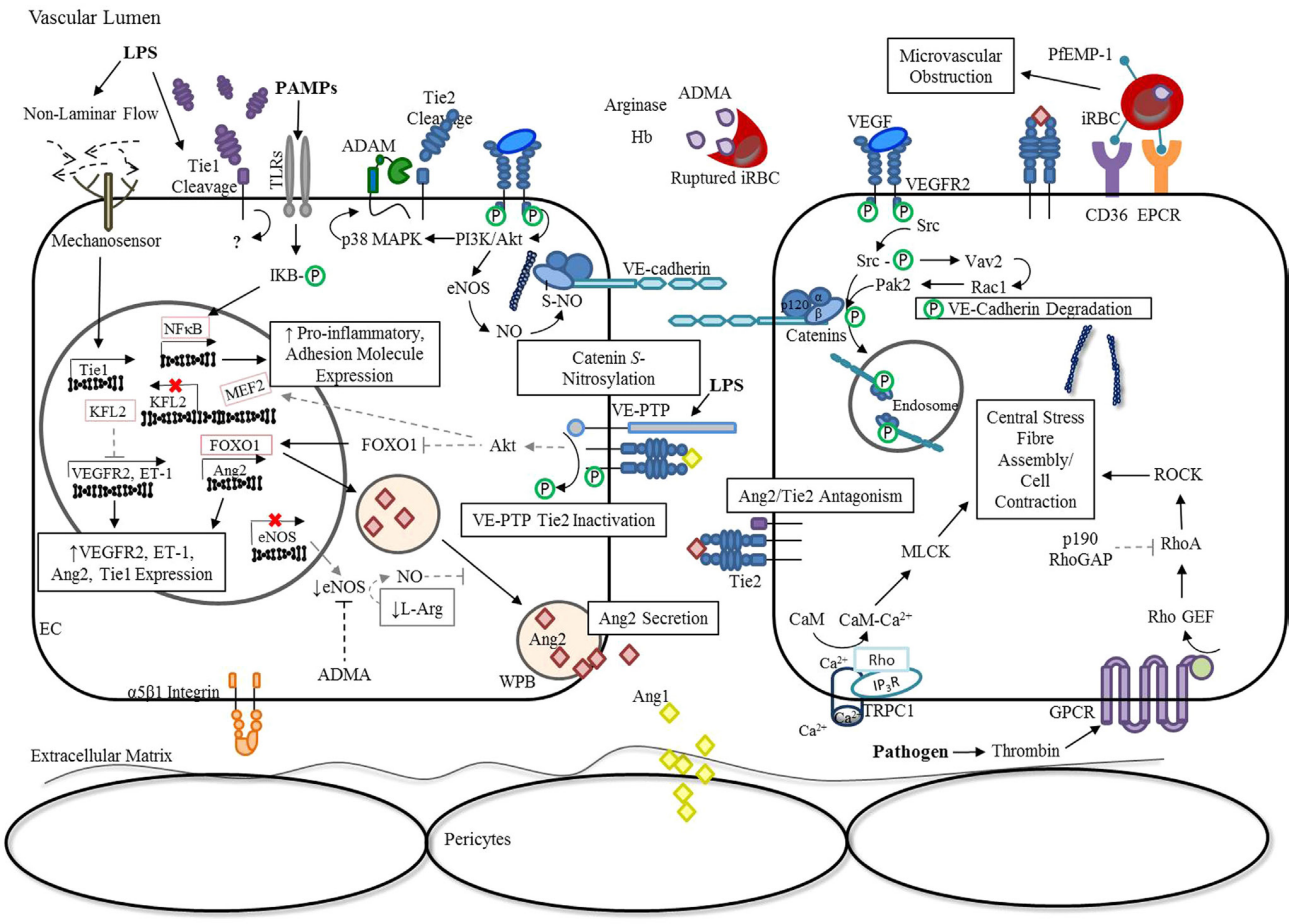
Endothelial activation: dysregulation of Ang/Tie signaling during severe infection. In response to inflammation, ECs release Ang2 from WPB into the vascular lumen, tipping Ang2:Ang1 ratios in favor of Ang2 (21). The release of ADMA, arginase, and hemoglobin from ruptured *Plasmodium falciparum* infected erythrocytes additionally decrease the availability of NO, enhancing WPB exocytosis (61, 62). Simultaneously, the presence of infection or TNF-α leads to Tie1 inactivation by ectodomain cleavage, thereby reducing the agnostic activity of Ang1 and promoting the antagonistic action of Ang2 (14, 15). Under conditions of hypoxia, such as an infection-induced reduction in laminar flow, VE-PTP expression is up-regulated (43), and a negative feedback loop is triggered that limits Tie2 activation by enhancing its association with VE-PTP (42). Interestingly, the transcription of Tie1 is also up-regulated in response to reduced laminar flow, despite ectodomain cleavage, suggesting an unknown role for the intracellular tyrosine kinase domain in signaling under these conditions (63). The resulting inactivation of Tie2 during infection and/or inflammation promotes FOXO1 transcriptional activity, thereby increasing Ang2 expression (15, 18, 20). The reduction in KFL2 expression associated with Tie2 inactivation stimulates VEGF-induced monocyte adhesion and vascular permeability (27, 64, 65). Furthermore, increased VEGFR2-signaling during infection leads to the activation of downstream pathways, such as PI3K/Akt and Src. Consequently, p38 MAPK-dependent activation of the protease, ADAM-15, induces Tie2 shedding and prevents its downstream signaling (66). In parallel, VEGFR2 activation of eNOS and Src further disrupt adherens junction complexes through *S-*nitrosylation of β-catenin (67) and phosphorylation of VE-cadherin (68, 69), respectively. Pathogens can indirectly lead to the activation of RhoA through GPCRs (70), for example, thrombin binding of protease-activated receptors (71, 72), to promote the formation of actin stress fibers that increase centripetal tension throughout the cytoskeleton (73). Furthermore, limited Tie2 activation during infection prevents p190RhoGAP inhibition of RhoA (35), and subsequent coupling of IP_3_R and TRPC1 to form a Ca^2+^ channel in the plasma membrane (74). This rise in intracellular Ca^2+^ leads to CaM-dependent activation of MLCK to further support EC contraction (35, 75). Lastly, TLR activation by PAMPs triggers the downstream activation of NF-κB to induce the expression of pro-inflammatory cytokines and adhesion molecules (76). This generates a positive feedback mechanism for endothelial destabilization, until infection and the associated inflammatory response are resolved (77). Abbreviations: ADAM-15, disintegrin and metalloproteinase domain-containing protein 15; ADMA, asymmetric dimethylarginine; Ang1, angiopoietin-1; Ang2, angiopoietin-2; Ca^2+^, calcium; CaM, calmodulin; CD36, cluster differentiation 36; EC, endothelial cell; eNOS, endothelial nitric oxide synthase; EPCR, endothelial protein C receptor ET-1, endothelin-1; FOXO1, forkhead box protein O1; GPCR, G-protein-coupled receptor; iRBC, *Plasmodium*-infected red blood cell; IP_3_R, inositol triphosphate receptor; IQGAP, IQ motif containing GTPase activating protein; KLF2, Krüppel-like factor-2; LPS, lipopolysaccharide; mDia, mammalian diaphanous; MEF2, myocyte enhancer factor-2; miR-30, microRNA-30-5p; MLCK, myosin light-chain kinase; NF-κB, nuclear factor-κB; NO, nitric oxide; p190RhoGAP, p190Rho GTPase-activating protein; p38 MAPK, p38 mitogen-activated protein kinase; PAMPs, pathogen-associated molecular patterns; PI3K, phosphoinositide triphosphate kinase; Rac1, RAS-related C3 botulinum toxin substrate 1; Rap1, Ras-related protein 1; RhoA, Ras homolog gene family, member A; RhoGEF: Rho guanine nucleotide exchange factor; S-NO, *S-*nitrosylation; Src, proto-oncogene tyrosine-protein kinase; TRPC1, transient receptor potential channel-1; VE-cadherin, vascular endothelial-cadherin; VEGF, vascular endothelial growth factor; VEGFR2, vascular endothelial growth factor receptor 2; VE-PTP, vascular endothelial protein tyrosine phosphatase; WPB, Weibel-Palade Body; P, phosphorylation.

In addition, binding of Ang1 to Tie2 can also stimulate the association of vascular endothelial (VE)-protein tyrosine phosphatase (PTP) with the Tie receptor complex (42). Under conditions of hypoxia, such as that resulting from infection-induced reduction in laminar flow, VE-PTP expression is up-regulated (43), and a negative feedback loop is triggered to limit Tie2 activation (42, 44). As outlined in Figure 2, there are a number of mechanisms employed by ECs to modulate Ang/Tie2 signaling during infection-induced endothelial activation. Findings from our group (45–53) and many others (54–60) have shown that disruption of any of these components related to the Ang/Tie axis may result in endothelial dysregulation and microvascular leak, regardless of the microbial etiology.

Although beyond the scope of this review it should be noted that both coagulation and complement activation contribute to the course and outcome of sepsis. A connection between the Ang/Tie2 pathway and coagulation in sepsis was revealed in a proteomic analysis of septic patients with disseminated coagulation (DIC). Findings from this study demonstrated that changes in Tie2 signaling was an initiating event in septic DIC and, at least in a mouse model, restoring Tie2 activation was sufficient to mitigate thrombosis (78). Additionally, the anticoagulant, activated protein C (APC) has been shown to bind and activate Tie2, leading to improved endothelial barrier integrity (79). Still, little remains known about the interplay between complement activation and Ang/Tie2 pathways in sepsis; this is an area that requires further investigation.

## The Ang/Tie Pathway in Severe Bacterial Infections

The incidence of severe sepsis in the United States is estimated at 3 cases per 1,000 population (80, 81). Mortality due to sepsis is high at approximately 20–55% (80–85), estimates that are relatively stable over nearly the past decade (86). Despite a high burden of disease and improved application of management strategies (87), there is a lack of effective treatments specific for sepsis (5, 88).

Gram-negative and Gram-positive infections occur with similar frequencies in hospitalized patients (89), and both can trigger sepsis mediated in part by the production of exotoxins or the release of bacterial cell wall components into the systemic circulation (32). These microbial products modulate many host response pathways, including in the Ang/Tie axis. The suppression of Ang1/Tie2 signaling and the associated microvascular leak are common features of many severe infections including bacterial sepsis (54). Reduced Tie2 activation during sepsis is the result of several perturbations to the pathway, including decreased Tie2 and Ang1 expression (24, 54, 57, 90–92), generation of soluble Tie receptors (14, 15, 40, 41, 66, 93, 94), and the antagonistic activity of Ang2 (14, 15).

Preclinical models of sepsis that have been used to explore the mechanisms and consequences of Tie2 signaling have shown that Tie2 expression and phosphorylation are greatly reduced during systemic infection. Inhibition of Tie2 activation leads to the nuclear localization of FOXO1 and transcription of its target genes, resulting in increased microvascular permeability (14, 15, 57, 91, 92). Tie2 mRNA levels have been shown to decline in response to decreases in endothelial shear stress associated with severe infection in a nuclear factor (NF)-κB dependent manner (92, 95). In contrast, the downregulation of Tie2 protein on the EC surface is not mediated by NF-κB but rather by the proteolytic cleavage of the Tie2 extracellular domain (92). *In vitro* studies have demonstrated both constitutive and VEGF-stimulated Tie2 receptor cleavage result in the release of a 75-kDa soluble Tie2 (sTie2) protein (66, 94, 96). The cleavage of the Tie2 ectodomain prevents Ang/Tie2 signaling and the circulating sTie2 which is generated may then function as a ligand trap, binding, and further inhibiting Ang activity. Indeed, the intravenous administration of adenoviral vectors expressing sTie2 blocked Ang/Tie2 signaling in *Mycoplasma pulmonis-*infected mice (97). Notably, the presence of sTie2 has been documented *in vivo* (93, 98, 99) and its levels are significantly increased in septic versus non-septic Intensive Care Units (ICU) patients (100). However, recent evidence incorporating mathematical and *in vitro* experimental modeling suggests that the molar ratio of sTie2:Ang1 levels found in patients with severe sepsis would have little influence on Ang/Tie2 activation *in vivo* (101).

In addition to reducing Tie2 expression, bacterial infections may also alter the functional activation state of this receptor, contributing to a leaky microvascular phenotype. Murine models of sepsis demonstrate a significant decline in the phosphorylated Tie2:total Tie2 ratio following LPS administration (91). This may be due, at least partly, to the influence of Tie1 on Tie2 activation. As already discussed, under baseline conditions, Ang1 or Ang2 stimulation of Tie2 promotes its interaction with Tie1 to form a heteromeric complex that is translocated to areas of cell–cell contacts (14, 17, 102). Oligomerized Ang1 bridges Tie2 at cell junctions resulting in the formation of *trans*-associations with Tie2 that preferentially activate Akt and its downstream signaling pathways, maintaining vascular quiescence (17). However in the mouse model, both LPS challenge and *M. pulmonis* infection induce cleavage of the Tie1 ectodomain responsible for its interaction with Tie2, thereby reducing Tie2 activation (14, 15). During endotoxemia, Tie1 cleavage also promotes antagonistic Ang2 activity resulting in the suppression of Tie2 signaling. This restores FOXO1 activity and establishes a positive feedback loop whereby FOXO1-driven Ang2 expression promotes microvascular leak during infection (14, 15).

Interestingly in murine models, Gram-negative bacteria may increase Ang2 expression by a different mechanism than that of Gram-positive bacteria. LPS has been shown to increase Ang2 expression *via* nicotinamide adenine dinucleotide phosphate (NADPH) oxidase 2 signaling through the inhibition of NFκ-B kinase subunit β/NF-κB and mitogen-activated protein kinase (MAPK)/activator protein 1 pathways (103), as well as the histone/protein deacetylase, Sirtuins 3 (24). Although mice treated with Gram-positive cell wall components (peptidoglycan and lipoteichoic acid) also exhibit elevated levels of Ang2 (24, 104), a comparison of bacterial etiologies among septic ICU patients revealed that Ang2/Ang1 ratios were significantly higher among patients with Gram-negative than those with Gram-positive infections (105). Recent evidence suggests that increased circulating Ang2 in Gram-positive infections is not the result of *de novo* biosynthesis, but rather the stimulated secretion of intracellular storage pools in response to the binding of cell wall components to TLR2 (24). While mechanistic differences may account for the more robust Ang2 increase observed in Gram-negative bacterial infections, these findings require *in vivo* confirmation.

During sepsis, an increase in Ang1 or decrease in Ang2 levels can enhance survival in murine bacterial sepsis models (56, 90, 106, 107). The overexpression of Ang1 using an adenoviral construct expressing human Ang1 (rh-Ang1) was found to attenuate LPS-induced expression of endothelial adhesion molecules in mouse lungs and kidneys, resulting in decreased leukocyte infiltration into interstitial spaces and minimizing hemodynamic instability (106, 107). In these mice, endothelial nitric oxide synthase (eNOS) expression was preserved and inducible nitric oxide (NO) synthase activity was decreased, contributing to reduced microvascular permeability in major organs (106, 107). The corresponding anti-inflammatory and anti-permeability effects of overexpressing Ang1 ultimately resulted in reduced organ injury as well as enhanced survival in endotoxemic mice compared to their Fc-controls. Despite the observed protective effect, little remains known as to how elevated levels of Ang1 impact the innate immune response. However, a recent study demonstrated that the improved survival conferred by the administration of rh-Ang1 in a mouse model of cerebral malaria (CM) was independent of its direct effects on parasitemia as both mice receiving rh-Ang1 and the Fc control had comparable parasite burdens (45). These findings suggest that increasing Ang1 levels during severe infection does not impair the host’s ability to resolve infection.

The protective effect observed with Ang1 has also been described in a cecal ligation and perforation (CLP) model of mice with one functional Ang2 allele (*Ang2^±^*), suggesting Ang2 contributes to multi-organ dysfunction and death in sepsis (56). In contrast, compared to their wild-type littermates, *Ang2^−/−^* knockout mice developed acute kidney injury following LPS exposure (90). Interestingly, the complete loss of Ang2 was previously observed to result in developmental abnormalities in mouse vasculature (108) likely rendering *Ang2^−^*^/^*^−^* mice susceptible to LPS-induced kidney injury. Nevertheless, these pre-clinical studies suggest that increasing circulating Ang1 and reducing Ang2 are associated with improved endothelial function during bacterial sepsis.

Bacterial pathogens are an important cause of sepsis, at least in part, due to their ability to induce systemic microvascular dysfunction through their interactions with the Ang/Tie system. A detailed understanding of mechanisms that regulate this pathway, and the ways in which bacteria modulate Tie2 activity, may suggest intervention strategies to maintain endothelial quiescence and microvascular integrity during severe bacterial infections. Preclinical studies of Tie2 directed therapies are reviewed in a later section.

## Endothelial Dysfunction in Malaria Infection

Malaria is an acute infectious disease caused by parasitic protozoa of the genus *Plasmodium*. Severe malaria is a sepsis syndrome that was responsible for an estimated 429,000 deaths in 2016 (109). It is typically caused by *Plasmodium falciparum* and is a complex multisystem disease with cerebral involvement (CM) being the most severe manifestation. Despite treatment with intravenous artesunate, CM has a reported case fatality rate of 30% in adults and 18% in children, with approximately one-third of survivors left with long-term neurological and neurocognitive deficits (110–114). While the pathophysiology of severe malaria is incompletely understood, it is characterized by marked inflammation, oxidative stress, and endothelial dysfunction and microvascular leak associated with disruption of the Ang/Tie2 axis.

The interaction between parasitized red blood cells (RBCs) and the host endothelium is central to the pathobiology of malaria infection and the development of severe disease. *P. falciparum*-infected RBCs (iRBCs) express the *P. falciparum* erythrocyte membrane protein 1 (PfEMP-1) on their cell surface, which mediates binding of the iRBCs to host EC receptors, including intracellular adhesion molecule-1 (ICAM-1), cluster of differentiation 36 (CD36), endothelial protein C receptor (EPCR), and gC1qR (115–118). This cytoadhesion leads to organ-specific sequestration of iRBCs, resulting in microvascular obstruction, impaired perfusion, hypoxia and metabolic derangements (119), all of which can contribute to endothelial activation (37, 63, 120, 121).

Endothelial stability is mediated in large part by two distinct but inter-related pathways, the Ang/Tie2 axis and the NO biosynthetic pathway (discussed below). Similar to bacterial sepsis, Ang1 has been shown to play a protective role in the severe malaria models (45), while several human studies have demonstrated a positive correlation between increased circulating Ang2 levels and poor clinical outcomes in pediatric and adult malaria infections (49, 52, 61, 122–124). Increased levels of Ang2 have also been implicated in the pathogenesis of placental malaria and its associated adverse birth outcomes, including stillbirth and fetal growth restriction (125–127).

The murine model of experimental CM (ECM) recapitulates several features of human severe and CM (128–132), including Ang dysregulation (45, 133). Deletion of the Ang1 locus increased susceptibility to ECM, while restoring circulating Ang1 levels maintained blood–brain barrier (BBB) integrity and enhanced survival, demonstrating that Ang1 is required to stabilize the microvasculature and improve outcome (45). The pathophysiology of human CM is debated, but likely results from both parasite and host determinants (134–138). Similar to ECM, recent human MRI data has provided evidence that BBB dysfunction and microvascular leak may contribute to BBB breakdown and cerebral edema in both adult and pediatric patients with CM (139, 140). In both pre-clinical and clinical studies, higher levels of Ang1 are associated with better outcomes, whereas higher levels of Ang2 correlate with disease severity and mortality. Thus, interventions that enhance Ang1 and/or Tie2 expression and activation may be beneficial in reducing malaria-associated adverse outcomes.

Nitric oxide is produced by a family of NO synthases (NOS) that use l-arginine as a substrate and the cofactor tetrahydrobiopterin. The rupture of iRBCs releases free hemoglobin, arginase, and contributes to the generation of asymmetric dimethylarginine (ADMA) in the circulation (Figure 2). These compounds collectively limit NO bioavailability *via* several mechanisms. Hemoglobin does so by reacting with NO, converting it to a biologically inactive nitrate. Proteolysis of erythrocyte proteins releases ADMA (an endogenous inhibitor of NOS) as well as arginase and converts arginine to ornithine, limiting the pool of arginine available for NO production (62, 141). Reduced levels of bioavailable NO lower the threshold for cytokine-induced EC activation and exocytosis of vWF and Ang2 from WPBs (21). Therefore, malaria infection fosters microenvironment conditions that facilitate the release of Ang2 and promote the switch from a quiescent to an activated endothelial phenotype. Interventions that improve NO bioavailability may be promising candidates for the treatment of severe malaria [discussed in Ref. (142–144)].

Less is known about the role of the Tie2 component of the pathway in malaria infection but reduced expression of Tie2 is observed in the lungs of malaria-infected mice (54) and increased levels of circulating sTie2 are observed in human severe malaria (48). These observations support the hypothesis that therapeutics that increase Tie2 expression may restore endothelial quiescence and reduce the risk of ALI and acute respiratory distress syndrome (ARDS) in human malaria infection (145). Ang/Tie2 targeted therapeutics in the treatment of malaria are discussed later in this review.

## Bench to Bedside: The Ang/Tie Pathway Components as Biomarkers of Life-Threatening Infections

Septic shock is the quintessential state of systemic endothelial dysfunction and microvascular leak. Over the last two decades, multiple studies of adult and pediatric populations have consistently shown that sepsis is marked by decreased levels of circulating Ang1 and increased levels of Ang2 [reviewed in Ref. (146–148)] and that the Ang2/Ang1 ratio can risk-stratify patients with critical illness. Notably, increased levels of Ang2 or a higher Ang2/Ang1 ratio predict mortality in septic patients (50, 53, 56, 100, 149–163). Among critically ill patients admitted to ICUs, plasma levels of Ang2 or the Ang2/Ang1 ratio increased across the spectrum of patients with sepsis and septic shock independent of the infecting pathogen (53, 56, 60, 150, 153, 155, 156, 158, 160, 161, 163–168). Ang2 levels correlated with surrogates of disease severity, including markers of tissue hypoperfusion, such as serum lactate (56, 60, 151, 155, 161), kidney injury (151, 154, 161), hepatic dysfunction (151), coagulopathy (151, 152), and markers of systemic inflammation (56, 60, 167). Ang2 levels were also associated with other clinical correlates of disease severity including Acute Physiology and Chronic Health Evaluation II (APACHE II) scores (56, 155, 161, 167), ICU length of stay (60), bacteremia (159), positive fluid balance (151, 162), need for corticosteroid support (151), and measures of organ failure (50, 53, 56, 60, 151–155, 160, 161, 164, 167, 169). Importantly, Ang2 levels measured early in the course of sepsis, including within 24 h of symptoms onset (158, 163, 166, 168), presentation to an Emergency Department (56, 150), and admission to ICU (50, 53, 60, 155, 157, 159, 160, 162, 164, 165), were associated with disease severity and predicted hospital mortality.

The circulating mediators of Tie2 signaling are particularly valuable in predicting outcomes of lung injury, likely owing to the high level of Tie2 expression in pulmonary vascular endothelium (147). In the lungs, Ang2 contributes to microvascular leak leading to pulmonary edema, ALI, and ARDS (55, 163, 168–173) [reviewed in Ref. (174)]. Elevated levels of circulating Ang2 not only correlate with disease severity, but also predict the degree of pulmonary microvascular leak (100, 163), duration of mechanical ventilation (163, 175), the partial pressure arterial oxygen to fraction of inspired oxygen (PaO_2_/FiO_2_) ratio (161–163, 168), and mortality (163, 169, 172, 173, 176, 177). Ang2 levels were a strong predictor of death in infection-mediated ARDS (149), an association that holds regardless of the inflammatory trigger.

Ang1 and Ang2 proteins have been evaluated as potential biomarkers of malaria disease severity and mortality, with Ang2 levels predicting not only in-hospital but also post-discharge mortality in children with severe malaria (46). Increased circulating levels of circulating Ang2 and decreased levels of Ang1 are associated with both severe *P. falciparum* (48, 49, 52, 122–124, 141, 178–183) and severe *Plasmodium vivax* infections (184–186) [reviewed in Ref. (187)]. Furthermore, the degree of Ang derangement correlates with the severity and outcome of *P. falciparum* infection (48, 49, 61, 122, 141, 181–184, 188), including anemia, jaundice, hypoglycemia (180), kidney injury (178, 180, 182), respiratory distress (181), CM (48, 52, 122, 178, 180, 189), and death (48, 49, 52, 61, 141, 180, 182, 189–191). Incorporating Ang2 concentrations into clinical scoring tools significantly improved the prediction accuracy of the models for mortality (48). Moreover, circulating Ang2 levels were informative in monitoring response to therapy and were predictive of short and long-term mortality (61, 122, 192).

## Therapeutic Interventions Targeting the Ang/Tie Axis

The observation that the Ang/Tie2 pathway contributes to disease pathobiology and that circulating ligands of Tie2, Ang1, and Ang2 can risk-stratify critically ill patients suggests that this pathway is a therapeutic target to prevent microvascular leak associated with sepsis. Off target effects of corticosteroids and HMG-CoA reductase inhibitors, both of which can reduce the severity of critical illness (193–198), have been shown to modulate Ang1 and Ang2 levels (199, 200) [reviewed in Ref. (36, 201, 202)]. Ang/Tie2-directed anti-angiogenic pharmacotherapies are in preclinical and clinical trials for the treatment of several malignancies and neovascular eye diseases [reviewed in Ref. (203)]; however, the development of adjunctive therapies for the management of sepsis and other critical illnesses associated with microvascular dysfunction have lagged behind. To date, pre-clinical studies with interventions that have stabilized the Tie2 receptor provide evidence that targeting this pathway may enable precision medicine approaches to improve outcomes of severe infections in humans [Table 1; reviewed in Ref. (146, 148)].

**Table 1:** Ang/Tie2-targeted therapies in pre-clinical studies of sepsis.

Compound(s)	Description	Pre-clinical studies/model
AdhAng1/rAAV.ANG1/AdAng1	Adenovirus construct expressing rh Ang1	Mouse—endotoxemia (106, 204) Mouse—ECM (45)

rh-Ang1	Commercial rh Ang1 protein (R&D Systems)	Mouse—endotoxemia (35) Mouse—Gram-negative sepsis (205)

COMP-Ang1	Adenovirus expressing rh-Ang1 variant: N-terminal is replaced with short coiled-coil domain of COMP for increased stability, solubility and Tie2 activating potency over rh-Ang1	Mouse—endotoxemia (107)

MAT.Ang1	rh-Ang1 variant: central coiled-coil N-terminal of Ang1 is replaced with short coiled-coil domain of matrilin for increased stability and solubility over rh-Ang1	Mouse—sepsis (58)

BOWAng1	rhAng1 variant: C-terminal fibrinogen-like domain of Ang1 protein fused to human IgG Fc fragment, engineered to tetramer conformation for optimal Tie2 phosphorylation	Mouse—ECM (45)

ANGPT1	Human Ang1 gene plasmid transfected into syngeneic MSCs for engraftment into injured pulmonary vasculature	Mouse—endotoxemia (206, 207)

CDDO-EA	Synthetic oleanane triterpenoid, activator of Nrf2. Increased Ang1 and decreased Ang2 levels in plasma, and reduced cerebrovascular leak in ECM model	Mouse—ECM (208)

Rosiglitazone	PPAR-γ agonist increased Ang1 levels in brains of ECM models	Mouse—ECM (133)

LC10, LOC06, ABA	Selective anti-Ang2 antibodies inhibit Ang2 binding to Tie2	Mouse—polymicrobial sepsis (24, 204)

ABTAA	Ang2 clustering converts antibody into Tie2 activating ligand	Mouse—endotoxemia, Gram-positive bacteremia, polymicrobial sepsis (24)

Angpt-2 siRNA	Ang2 siRNA highly specific for pulmonary endothelium, reduced Ang2 expression in murine lung tissue and resulted in increased Tie2 phosphorylation	Mouse—endotoxemia, polymicrobial sepsis (209)

rh-Ang2	Commercial rh Ang2 protein (R&D Systems)	Mouse—Gram-negative sepsis (205) Rabbit—Gram-negative sepsis (210)

AKB-9778	Small molecule inhibitor of VE-PTP; promotes Tie2 activation	Mouse—endotoxemia (33) Mouse—stroke/BBB permeability (211) Mouse—choroidal neovascularization and ischemic retinopathy (43, 212)

Vasculotide	Synthetic tetrameric polyethylene glycol-clustered Tie2 agonist	Mouse—polymicrobial sepsis (213) Mouse—influenza infection (214)

*Therapeutic agents that target Tie2 by augmenting Ang1 levels, inhibiting Ang2, or promoting Tie2*.

*Abbreviations: ABA, Ang2-blocking antibody; ABTAA, Ang2-binding and Tie2 agonist antibody; Angiopoietin (Ang), CDDO-EA, 2-cyano-3,12-dioxooleana- 1,9(11)-dien-28-oic acid ethyl amide; COMP, cartilage oligomeric matrix protein; ECM, experimental cerebral malaria; Fc, IgG, immunoglobulin; MAT, matrilin; nrf2, nuclear factor-like 2; siRNA, small interfering RNA; rh, recombinant human; VE-PTP, vascular endothelial protein tyrosine phosphatase*.

Studies using murine models of sepsis have demonstrated that therapeutic compounds that augment Ang1 expression can attenuate many of the adverse outcomes associated with endotoxemia. Increased Ang1, driven by adenovirus-mediated rh gene delivery prior to an LPS challenge, preserved eNOS activity in lung tissue and reduced lung injury, prevented up-regulation of cellular adhesion molecules, improved hemodynamics, and reduced mortality (106, 204). A more potent and stable Tie2 phosphorylating molecule, cartilage oligomatrix protein (COMP)-Ang1 (215) similarly prevented adhesion molecule expression and conferred renal protection in the sepsis model (107). Although these findings establish the protective benefit of sustaining Tie2 phosphorylation through Ang1 treatment in sepsis, the use of an adenoviral delivery vector is problematic for translation to human therapy. Subsequent studies demonstrated that rh Ang1 (rh-Ang1) delivered systemically to mice undergoing cecal ligation and perforation (CLP) stabilized endothelial barrier function, preventing pulmonary capillary leak, and decreased leukocyte infiltration into both lungs and kidneys by suppressing ICAM-1 expression (35). Despite the short half-life of rh-Ang1 (216), treatment was able to avert multi-organ dysfunction and increased survival following CLP (59). Matrilin-1-Ang1 (MAT.Ang1) was developed as a stable Ang1 variant amenable to direct intravenous administration; it too stabilized the endothelium in the setting of LPS-induced endotoxemia (58).

Ang1 therapy has also been shown to mitigate the adverse sequelae of severe malaria infection. Using BowAng1, a rh Ang1 protein capable of phosphorylating Tie2 (217), it was demonstrated that its addition to artesunate therapy preserved the integrity of the BBB and improved survival in a murine model of CM, even when administered during the late stage of infection (45). Other therapeutic agents that increase Ang1 expression also prevent BBB leak and improve survival in ECM. Mice treated with the ethyl amide of a synthetic oleanane triterpenoid, 2-cyano-3,12-dioxooleana-1,9(11)-dien-28-oic acid (CDDO-EA), had increased Ang1, reduced Ang2 and Ang2:Ang1 ratio, and this was associated with improved BBB integrity (208). Similarly, treatment of malaria infected mice with the PPAR-γ agonist rosiglitazone in combination with artesunate at the onset of neurological symptoms achieved higher plasma and brain levels of Ang1 and a lower Ang2:Ang1 ratio compared to mice treated with artesunate alone. Furthermore, these mice had enhanced BBB integrity, improved survival and better cognitive and motor outcomes than mice treated with anti-malarials alone (133). Rosiglitazone has entered human clinical trials. To date, a randomized clinical trial of rosiglitazone in young adults with uncomplicated malaria showed reduced levels of pro-inflammatory mediators, a lower Ang2:Ang1 ratio, and higher levels of brain-derived neurotrophic factor, a protein involved in neuronal survival and proliferation (133, 218). A phase IIa study has shown rosiglitazone to be safe and well tolerated in pediatric patients with uncomplicated malaria and is currently being tested in a phase IIb trial in children with severe malaria (219).

Other novel modes of augmenting Ang1 expression are currently in preclinical trials. Preliminary work utilizing cell-based therapy has demonstrated that mesenchymal stem cells (MSCs) transfected with Ang1 are able to engraft the pulmonary endothelium damaged during sepsis, preserve pulmonary endothelial integrity, and ameliorate ALI/ARDS (206, 207).

In contrast to the constitutive expression of Ang1, Ang2 is relea-sed in response to infectious triggers with considerable dynamic range (56, 168), making this molecule an appealing target for pharmacologic inhibition in sepsis. In preclinical trials, lung-targeted small interfering RNA (siRNA) against Ang2 delivered both pre- and post-sepsis induction reduced pulmonary inflammatory cytokine levels, ICAM-1 expression, neutrophil organ infiltration, and overall disease severity while improving survival (209). Functional inhibition of Ang2/Tie2 binding using anti-Ang2 antibodies decreased rates of hemodynamic shock and mortality in murine sepsis and ARDS models (24, 204).

Furthermore, the novel Ang2-binding and Tie2-activating antibody (ABTAA) utilizes a mechanism through which Ang2 clustering converts the antibody-antigen cluster into a Tie2 activating ligand, thus allowing simultaneous Ang2 inhibition and Tie2 activation (24). When compared to the conventional anti-Ang2 antibody, ABTAA conferred increased protection against microvascular dysfunction, end-organ damage, and mortality in CLP, endotoxemia, and *Staphylococcus aureus* models of sepsis (24). In combination with broad-spectrum antibiotics (imipenem/cilastatin), ABTAA improved survival to 70%, compared to 20% survival in animals treated with antibiotics alone in the CLP model (24). Seemingly paradoxically, studies in murine and rabbit models of pyelonephritis and sepsis found that rh-Ang2 administration prolonged survival in Gram-negative sepsis (205, 210). Notably, these studies did not assess Tie2 phosphorylation status, leaving the mechanism of Ang2-mediated survival in these models unclear.

In light of these findings, further analyses have been performed examining the role of simultaneous Ang2 inhibition and Tie2 activation in vascular protection during sepsis (24). When treated with the antibody ABTAA, mice with high-grade CLP had significantly improved survival rates (40%) compared to the conventional Ang2-blocking antibody (ABA; 13%). These findings were further extended to two other sepsis models used in this study: endotoxemia (rate of survival increase: 63% ABTAA vs 33% ABA) and *S. aureus* bacteremia (rate of survival increase: 55% ABTAA vs. 9% ABA). In these models, it was observed that ABTAA ameliorated endotoxemic and CLP-induced sepsis by preserving endothelial glycocalyx and microvascular integrity of major organs (24). Taken together, these studies underline the importance of Tie2 activation in ameliorating the progression of sepsis and demonstrate that solely blocking Ang2 is insufficient for preserving endothelial integrity during severe bacterial sepsis.

In addition to targeting the Tie2 receptor through Ang1 and Ang2, several other agents have been used to maintain Tie2 phosphorylation in animal models of sepsis. For example, pharmacologic inhibition of the Tie2 phosphatase, VE-PTP, with AKB-9778 stabilized the pulmonary endothelium following LPS administration in mice (33), offering another potential mechanism to modulate the activity of Tie2 in sepsis. This compound has already been used in human trials to treat diabetic macular edema and ocular neovascularization (43, 212). Alternatively, Vasculotide, a synthetic polyethylene glycol-clustered Tie2 agonist, has been shown to sustain Tie2 activation *in vivo*. Its administration both pre- and post-CLP reduced end-organ dysfunction and mortality in the murine abdominal sepsis model (155). Vasculotide administration also preserved pulmonary endothelial barrier function and survival following murine infection with several strains of influenza. Importantly, the protective effect was realized even with therapy delayed up to 72 h after infection, conditions similar to typical septic patient presentations (214). Unlike interventions that manipulate Ang1 or Ang2 expression, Vasculotide is highly specific for the Tie2 receptor and does not displace Ang1 or Ang2. As such, Vasculotide may avoid interfering with off-target effects of Ang1 during the dynamic host sepsis response.

Although the pathophysiology Tie2-mediated vascular dysfunction in sepsis remains incompletely understood, the effects of these therapies in preclinical sepsis models warrant further investigation to develop human Tie2-directed therapies.

## Conclusion

The Ang/Tie2 axis plays an essential role in maintaining endothelial barrier stability and its disruption during systemic infection contributes to the pathologic cascade that culminates in end-organ failure and death. In addition to its mechanistic role in the pathobiology of sepsis, components of the Ang/Tie2 system can function as prognostic biomarkers of disease severity and outcomes, and potentially serve as important therapeutic targets in the management of sepsis.

The dysregulation of Ang/Tie2 signaling is “pathogen agnostic” and appears to represent a final common pathway in many different types of microbial infections, including bacterial and parasitic processes described in this review. As such, therapeutic interventions to restore Tie2 activity may be useful in the early management of serious infections where there is a high degree of diagnostic uncertainty. Furthermore, use of Ang/Tie2 adjunctive therapy in sepsis may confer protection against the collateral systemic damage that results in significant morbidity and mortality. The pathophysiology Tie2-mediated microvascular dysfunction in sepsis remains incompletely understood, but the findings from preclinical sepsis models warrant further investigation with the aim of developing human Tie2-directed therapies to improve outcomes of life-threatening infections.

## Author Contributions

AL, MR-G, JW, VC, and KK conceived the ideas for preparing this review. AL, MR-G, JW, VC, and KK contributed to the writing, editing, and approval of the final review.

## Conflict of Interest Statement

The authors declare that the research was conducted in the absence of any commercial or financial relationships that could be construed as a potential conflict of interest.

## Abbreviations

ABIN2, A20-binding inhibitor of nuclear factor-κB-2; ABA, Ang2-blocking antibody; ABTAA, Ang2-binding and Tie2-activating antibody; ADAM-15, disintegrin and metalloproteinase domain-containing protein 15; ADMA, asymmetric dimethyl arginine; Ang, angiopoietin; ARDS, acute respiratory distress syndrome; BBB, blood brain barrier; BDNF, brain-derived neurotrophic factor; Ca^2+^, calcium; CaM, calmodulin; CD36, cluster of differentiation 36; CDDO-EA, 2-cyano-3,12-dioxooleana- 1,9(11)-dien-28-oic acid ethyl amide; CM, cerebral malaria; COMP-Ang1, cartilage oligomatrix protein Ang1; EC, endothelial cell; ECM, experimental cerebral malaria; EPCR, endothelial protein C receptor; ER, endoplasmic reticulum; ET-1, endothelin-1; FOXO1, forkhead box protein O1; GPCR, G-protein-coupled receptor; PAMP, pathogen-associated molecular pattern; ICAM-1, intracellular adhesion molecule-1; ICU, intensive care unit; IP_3_R, inositol triphosphate receptor; IQGAP, IQ motif containing GTPase activating protein; KLF2, Krüppel-like factor-2; iRBC, infected red blood cell; LPS, lipopolysaccharide; MAT, matrilin; mDia, mammalian diaphanous; MEF2, myocyte enhancer factor-2; miR-30, micro ribonucleic acid-30-5p; MLCK, myosin light-chain kinase; NF-κB, nuclear factor-κB; NO, nitric oxide; NOS, nitric oxide synthase; eNOS, endothelial nitric oxide; NFR2, nuclear factor erythroid 2-related factor 2; p38 MAPK, p38 mitogen-activated protein kinase; p190RhoGAP, p190 Rho GTPase-activating protein; PfEMP-1, *Plasmodium falciparum* erythrocyte membrane protein-1; PI3K, phosphoinositide triphosphate kinase; Rac1, RAS-related C3 botulinum toxin substrate 1; rh-Ang1, recombinant Ang1; Rap1, Ras-related protein 1; RBC, red blood cell; rh, recombinant human; RhoA, Ras homolog gene family, member A; RhoGEF, Rho guanine nucleotide exchange factor; SOFA, sequential organ failure assessment; Src, proto-oncogene tyrosine-protein kinase; TRPC1, transient receptor potential channel-1; VE-cadherin, vascular endothelial-cadherin; VEGF, vascular endothelial growth factor; VEGFR, vascular endothelial growth factor receptor; VE-PTP, vascular endothelial protein tyrosine phosphatase; WPB, Weibel-Palade bodies; p38 MAPK, p38 mitogen-activated protein kinase; PPAR, peroxisome proliferator-activated receptor.
